# Immune Checkpoint Pathway Expression in Lymphocyte Subpopulations in Patients with Common Variable Immunodeficiency and Chronic Lymphocytic Leukemia

**DOI:** 10.3390/cancers15215184

**Published:** 2023-10-28

**Authors:** Paulina Mertowska, Sebastian Mertowski, Konrad Smolak, Aleksandra Kita, Gabriela Kita, Katarzyna Guz, Marcin Pasiarski, Ewelina Grywalska

**Affiliations:** 1 Department of Experimental Immunology, Medical University of Lublin, 20-093 Lublin, Poland; paulina.mertowska@umlub.pl (P.M.);; 2 Student Research Group of Experimental Immunology, Medical University of Lublin, 20-093 Lublin, Poland; 3 Department of Immunology, Faculty of Health Sciences, Jan Kochanowski University, 25-317 Kielce, Poland; 4 Department of Hematology, Holy Cross Cancer Centre, 25-734 Kielce, Poland

**Keywords:** CTLA-4, PD-1, PD-L1, CD86, CD200, CD200R, common variable immunodeficiency, chronic lymphocytic leukemia, immunodeficiency

## Abstract

**Simple Summary:**

Our research explores the realm of immune cells to better understand patients with chronic lymphocytic leukemia (CLL) and common variable immunodeficiency (CVID). By examining immune checkpoints such as PD-1/PD-L1, CTLA-4/CD86, and CD200R/CD200 in different blood lymphocyte subpopulations, we aim to extract valuable insights that may improve not only our understanding of these diseases but also contribute to improving treatment conditions and the occurrence of subsequent complications and the development of hematological cancers. We hope that the knowledge about the role of these signaling pathways in the development and progression of these two diseases will allow us to develop modern and personalized diagnostic and therapeutic strategies, which in the future may allow monitoring the immune system of patients with CVID and CLL.

**Abstract:**

This study aims to gain a deeper understanding of chronic lymphocytic leukemia (CLL) and common variable immunodeficiency (CVID) by studying immune cells and specific immune checkpoint signaling pathways. The analysis of the percentage of selected immune points and their ligands (PD-1/PD-L1, CTLA-4/CD86, and CD200R/CD200) on peripheral blood lymphocyte subpopulations was performed using flow cytometry, and additional analyses determining the serum concentration of the above-mentioned molecules were performed using enzyme immunoassay tests. The obtained results indicate several significant changes in the percentage of almost all tested molecules on selected subpopulations of T and B lymphocytes in both CVID and CLL patients in relation to healthy volunteers and between the disease subunits themselves. The results obtained were also supported by the analysis of the serum concentration of soluble molecules tested. By uncovering valuable insights, we hope to enhance our comprehension and management of these conditions, considering both immunodeficiencies and hematological malignancies. Understanding the role of these signaling pathways in disease development and progression may lead to the development of modern, personalized diagnostic and therapeutic strategies. Ultimately, this knowledge may enable the monitoring of the immune system in patients with CVID and CLL, paving the way for improved patient care in the future.

## 1. Introduction

Hematologic malignancies refer to cancers originating in the blood, bone marrow, and lymphatic system. They are primarily characterized by the uncontrolled growth and division of abnormal cells in these tissues. Currently, the main types of hematological cancers include leukemia, lymphoma, and multiple myeloma [1,2,3]. Literature data highlight the link between hematological malignancies and the development of immunodeficiencies, a condition in which the immune system is weakened, leading to increased susceptibility to infections and other diseases [4,5,6,7]. Immune deficiencies can be primary-PID (congenital or inherited) or secondary-SID (acquired due to other factors such as infections, medications, or certain medical conditions) [8,9]. Research shows that some immunodeficiency disorders can increase the risk of developing hematologic cancers, and people with hematologic cancers may experience immunodeficiency as a result of the disease or its treatment [9].

Chronic lymphocytic leukemia (CLL) is one of the most common types of hematological malignancies (belonging to the category of lymphoid malignancies) in which SID develops. This disease mainly focuses on the impaired functioning of B lymphocytes and is characterized by the progressive accumulation of these atypical, fully undeveloped lymphocytes in the circulatory system, bone marrow, and lymphoid tissues. These abnormal cells do not function properly and displace healthy blood cells, leading to various complications [10,11,12]. CLL can be asymptomatic in its early stages, and the disease can be discovered incidentally during routine blood tests. As the disease progresses, symptoms may include enlarged lymph nodes, fatigue, frequent infections, night sweats, weight loss, and anemia. Treatment for CLL varies depending on the stage of the disease and the patient’s overall health [13,14]. Some common treatment options include watchful waiting for asymptomatic early-stage CLL, chemotherapy, targeted therapies, immunotherapy, and stem cell transplantation for more advanced cases. The choice of treatment will depend on a variety of factors, including the patient’s age, overall health, and genetic markers for the leukemia cells [15,16,17].

PID represents a diverse collection of inherited disorders characterized by irregular immune function, resulting in heightened vulnerability to infections and/or imbalanced immune responses [18,19]. Moreover, certain PIDs are more inclined toward developing malignancies. Research involving patients registered in the United States Immune Deficiency Network (USIDNET) from 2003 to 2015 indicated a 1.42-fold elevated relative risk of cancer compared to the age-matched population in the Surveillance, Epidemiology, and End Results Program (SEER) database [20,21]. The majority of these cancers were blood-related, primarily lymphoid, and linked to the specific cell types affected by PID [21]. Common variable immunodeficiency (CVID) stands as the most prevalent clinically apparent primary immunodeficiency, constituting a diverse range of disorders, all characterized by the primary failure to produce essential antibodies [22,23,24]. Alongside unconventional infections, non-infectious indications like autoimmunity, autoinflammation, and widespread lymph node enlargement might precede the manifestation of evident immunodeficiency and take precedence in the CVID presentation [25,26,27,28]. A recent comprehensive analysis exhibited an 8.6% occurrence of malignancies within CVID cases. Predominant among these malignancies, which also display an elevated frequency among CVID patients, are lymphomas and gastric cancer [25,26,27,28,29,30].

In the course of both these groups, there are significant disorders of the immune system, which affect not only its proper functioning but also the maintenance of immune homeostasis, the disturbance of which may result in the development of autoimmunity or cancer [31]. Research in recent years points to immunological checkpoints as key elements of the ongoing deregulation. Immune checkpoints are molecules or receptors on immune cells that regulate the immune response to maintain self-tolerance and prevent excessive immune activation that can lead to autoimmune reactions [32,33,34]. These checkpoints play a key role in balancing the activity of the immune system, enabling it to respond to pathogens and cancer cells while avoiding damage to healthy tissues. However, cancer cells can sometimes use these checkpoints to avoid being attacked by the immune system [35,36,37]. They express certain proteins that interact with checkpoint receptors on immune cells, effectively suppressing the immune response against cancer cells. This mechanism is one way through which cancer can bypass the immune system and continue to progress [38,39,40].

Therefore, the aim of this publication was to evaluate three immunological checkpoints of their ligands: PD-1 (CD279)/PD-L1 (CD274), CTLA-4 (CD152)/CD86, and CD200R/CD200 in the course of immunodeficiencies, on the example of CLL and CVID. For this purpose, we conducted a series of analyses to assess selected parameters of the morphology, biochemistry, and immunophenotype of patients’ peripheral blood, with particular emphasis on the assessment of the percentage of the presence of the tested molecules on selected subpopulations of T and B lymphocytes. Their concentration of soluble forms in the serum was also analyzed. Based on the obtained data, we performed a correlation analysis of the examined immune system parameters with selected biological parameters and also analyzed the obtained results in terms of their diagnostic usefulness as potential biomarker molecules for recognizing and monitoring the development and progression of CLL and CVID.

## 2. Materials and Methods

### 2.1. Characteristics of Patients and Research Material

A total of 120 patients were included in the study: 40 people diagnosed with chronic lymphocytic leukemia (CLL), 40 people diagnosed with common variable immunodeficiency (CVID), and 40 healthy volunteers (HV) as a control group; also, at the same time, patients with CLL and CVID were newly diagnosed patients. All immunodeficient patients and HV were subject to the inclusion and exclusion criteria. Patient selection was performed by a physician experienced in Clinical Immunology, based on specific criteria: age ≥18 years; life expectancy ≥ 12 months; no immunosuppressive treatment within three months before study entry; and written consent to participate in the study. Criteria for exclusion of patients from the study: active viral, bacterial, or fungal infection; severe allergy; condition after allotransplantation of hematopoietic cells or internal organs; active malignancy or another autoimmune disease under treatment; the period of pregnancy or lactation; taking drugs that are in the phase of clinical trials; the presence of tumor metastases within the central nervous system; and mental illness. In addition, all patients were matched for age and sex: CVID (median age: 49.1; range: 27–70; 15 females and 25 males); CLL (median age: 48.2; range: 31–73; 16 females and 24 males); HV (median age: 48.8; range: 32–70; 17 women and 23 men). The research material consisted of 10 mL of peripheral blood sampled from the basilic vein into EDTA (Strasted) tubes (used for immunophenotypic analysis) and 5 mL of blood collected in a clot tube (to obtain serum for quantitative determination of soluble forms of the tested molecules).

The study protocol was positively evaluated by the Bioethics Committee at the Medical University of Lublin (KE-0254/276/2021).

### 2.2. Analysis of the Immunophenotype of Peripheral Blood

To analyze the lymphocyte immunophenotype in the peripheral blood using flow cytometry, a blood sample was taken and treated with a specific set of monoclonal human antibodies, including anti-CD45 Alexa Fluor 700, anti-CD4 BV421, anti-CD3 PerCp, anti-CD8 BV605, and anti-CD19 FITC, as well as anti-PD-1 (CD279) APC, anti-PD-L1 (CD274) PE, anti-CTLA-4 (CD152) PE, anti-CD86 APC, anti-CD200 PE, and anti-CD200R APC antibodies (Biolegend in San Diego, CA, USA). FMO controls were used for each antibody to assist in cytometric analysis. After the antibody staining process, red blood cells were removed using a lysing buffer (BD, Franklin Lakes, NJ, USA). The resulting cells were washed and evaluated using a CytoFLEX LX instrument (Beckman Coulter, Indianapolis, Brea, CA, USA). The Kaluza Analysis program (Kaluza Analysis v 2.1, Beckman Coulter, Indianapolis, Brea, CA, USA) was used for data analysis. The CytoFLEX LX flow cytometer underwent daily quality control using CytoFLEX Ready to Use Daily QC Fluorospheres reagents (Beckman Coulter, Indianapolis, Brea, CA, USA). An example analysis is presented in Supplementary Figure S1.

### 2.3. Evaluation of the Concentration of Soluble Forms of Immune Checkpoints and Their Ligands in the Serum

The immunoenzymatic evaluation of the concentration of all tested molecules was performed using serum collected from all patients. Tests were performed using commercially available kits according to the manufacturer’s instructions: Human CD200 ELISA Kit (sensitivity: 20 pg/mL; range: 24.58–6000 pg/mL) (Invitrogen, Waltham, MA, USA); Human CD200R ELISA Kit (Sensitivity: 11.89 pg/mL; Range: 46.88–3000 pg/mL) (Abcam, Cambridge, UK); Human CTLA-4 (CD152) ELISA Kit (Sensitivity: 0.13 ng/mL; Range: 0.16–100 ng/mL) (Invitrogen, Waltham, MA, USA); Human CD86 ELISA Kit (Sensitivity: 0.82 ng/mL; Range: 0.82–200 ng/mL) (Invitrogen, Waltham, MA, USA); Human PD-1 (CD279), ELISA Kit (Sensitivity: 1.14 pg/mL; Range: 2.34–150 pg/mL), (Invitrogen, Waltham, MA, USA); Human ELISA Kit PD-L1 (CD274), (Sensitivity: 0.6 pg/mL; Range: 4.69 ng/mL–0.300 pg/mL), (Invitrogen, Waltham, MA, USA). The measurement was performed using a VictorTM3 reader (PerkinElmer, Waltham, MA, USA).

### 2.4. Statistical Analysis of the Obtained Test Results

The obtained results were subjected to statistical analysis using Tibco Statistica 13.3 software in Palo Alto, CA, USA. The normality of the data distribution was assessed using the Shapiro–Wilk test. Differences between the groups were analyzed using the Kruskal–Wallis test, followed by Dunn’s post hoc test. For Dunn’s test, *p*-values were adjusted for multiple comparisons using the Bonferroni method. To explore relationships between pairs of variables, Spearman’s correlation coefficients were used. The diagnostic performance of the laboratory test was assessed using ROC curves for patient-related parameters. Visualizations of the data were created using GraphPad Prism (GraphPad Prism Software v. 9.4.1 (687), San Diego, CA, USA).

## 3. Result

### 3.1. Comparative Analysis of Infections and Selected Peripheral Blood Parameters (Morphology, Biochemistry, Immunophenotype) of Patients

The two diseases selected for the study represent two groups of immunodeficiencies: patients with CVID are classified as PID, while patients with CLL are classified as SID. The comparison of these two diseases may seem unnecessary at first glance, but considering that patients diagnosed with CVID have an increased risk of developing hematological cancers, including leukemia and lymphoma, the aim is to show the changes that may occur in the patients’ bodies and compare changes occurring in PID and SID. The general risk of cancer development in patients with CVID is estimated to fall within a range of 4% to 25%, and the projected incidence of malignancies is approximately 10%. Adult-onset CVID patients are at an increased risk for cancer compared to other age groups [25,26,27,28,29,30]. All patients (CVID and CLL) included in these analyses were newly diagnosed patients who met the detailed inclusion and exclusion criteria described in the Materials and Methods section and were not on any therapy before or at the time of this study. 

The number of infections requiring antibiotic therapy in the last 12 months before diagnosis was also assessed for all patients included in the study. This analysis showed that 97.5% of CVID patients and 95% of CLL patients required antibiotic therapy to control the infection. The most common infections reported were upper and lower respiratory tract infections, gastrointestinal infections, skin infections, and urinary tract infections. In patients with CVID, 90% of recruited patients had used antibiotics to combat upper and lower respiratory tract infections in the last 12 months; 65% of patients used antibiotic therapy for gastrointestinal infections, 25% for skin infections, and 27.5% for urinary tract infections. In CLL patients, antibiotic therapy was necessary for 77.5% of patients due to upper and lower respiratory tract infections, 52.5% due to gastrointestinal infections, 32.5% due to skin infections, and 22.5% due to urinary tract infections. The number of individual infections is presented in Supplementary Figure S2.

Due to the diversity of the disease entities included in this study, we first decided to analyze several important biological parameters, such as the analysis of the available results of peripheral blood morphology and the biochemistry of our patients. This analysis showed several statistically significant changes. CVID patients representing the PID group showed increased levels of white blood cells (WBCs) relative to healthy individuals, as well as decreased levels of lymphocytes (LYMs), neutrophils (NEUs), red blood cells (RBCs), hemoglobin (HGB), platelets (PLTs), and a characteristic decrease in immunoglobulin G (IgG) and IgA (Supplementary Table S1). Patients with CLL representing the group of patients with SID also showed numerous changes, including increased WBC and LYM, as well as decreased RBC, HGB, PLT, IgG, IgM, and IgA relative to healthy volunteers (Supplementary Table S1). Moreover, a detailed comparative analysis of these parameters between CVID and CLL patients showed a significant increase in WBCs, LYMs, NEUs, basophils (BASs), RBCs, HGB, PLTs, IgG, and IgA in SID patients relative to PID (Supplementary Table S1).

We also supplemented the basic analysis of peripheral blood parameters with an immunophenotype assessment, indicating the state of the immune system of our patients. This analysis provided a lot of valuable information about the immune system status of the recruited patients, which is extremely helpful in the diagnosis and monitoring of various disease states, including immunodeficiency or hematopoietic malignancies. Abnormalities or imbalances in certain populations of immune cells may indicate potential health problems or ongoing immune responses in the body. In our case, the obtained research data showed a number of significant changes in both studied patient populations, both in relation to HV and between each other. This may indicate disorders in the functioning of the normal immune response (Supplementary Table S2). In patients with CVID, compared to HV, we observe a slight decrease in the frequency of CD45+ leukocytes and CD3+ T lymphocytes, as well as an almost 30% decrease in the percentage of CD19+ B lymphocytes. We also noted significant differences in the percentage of CD4+ T lymphocyte subpopulations (a decrease of 38.02%). In addition, we also observe a reduced CD4+/CD8+ ratio. These observations are often a characteristic feature of the development of PID, including a decrease in the number of T lymphocytes and a decrease in the CD4 to CD8 ratio (this ratio is reduced even to <1), as well as a reduced number of B lymphocytes; these changes may indicate an impaired response of the body to all kinds of infections that often accompany PID, including patients with CVID. In patients with CLL, compared to HV, we observe a slight decrease in the frequency of CD45+ leukocytes and a significant decrease in CD3+ T lymphocytes (by 82.98%), as well as a decrease in the percentage of individual subpopulations of CD4+ T lymphocytes (by 87.34%) or CD8+ T lymphocytes (by 84.17%). A significant increase was noted in the percentage of B lymphocytes by 6.70 times compared to HV. The observed changes are also characteristic of this type of disease. The analysis of the obtained immunophenotype results between patients with PIS and SID also showed statistically significant changes in almost every analyzed parameter: a decrease in the percentage of CD3+ T lymphocytes in patients with CLL (5.42-fold), a decrease in the percentage of CD4+ and CD8+ T cells in CLL patients (by 4.89-fold and 6.60-fold, respectively), as well as an increase in the percentage of B cells in CLL patients (by 9.59-fold) (Supplementary Table S2). 

### 3.2. Comparative Analysis of Selected Subpopulations of Peripheral Blood Lymphocytes Showing Positive expression of the Tested Immunological Checkpoints and Their Ligands

**Figure 1:**
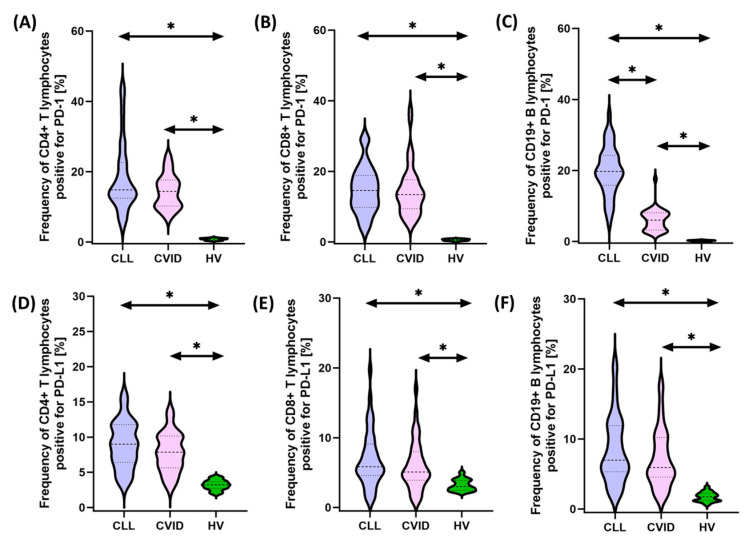
Schematic representation of the analysis of the percentage of selected subpopulations of T and B lymphocytes expressing positive PD-1 and PD-L1 among patients with CVID, CLL, and HV. (**A**) Percentage of CD4+ T cells positive for PD-1 expression; (**B**) percentage of CD8+ T cells positive for PD-1 expression; (**C**) percentage of CD19+ B cells positive for PD-1 expression; (**D**) percentage of CD4+ T cells positive for PD-L1 expression; (**E**) percentage of CD8+ T cells positive for PD-L1 expression; and (**F**) percentage of CD19+ B cells positive for PD-L1 expression. * statistically significant results. Abbreviations: CVID—common variable immunodeficiency; CLL—chronic lymphocytic leukemia; CD—cell differentiation antigen; PD-1—Programmed Cell Death Protein 1; PD-L1—Programmed Cell Death Ligand 1; and HV—healthy volunteers.

**Figure 2:**
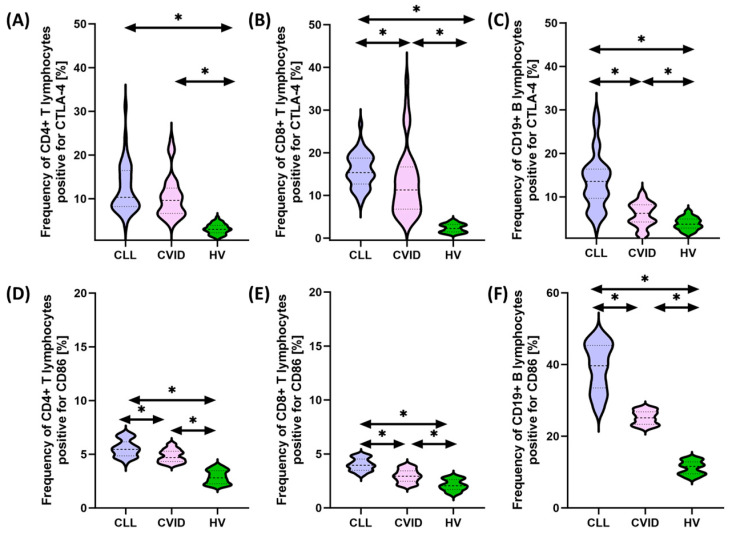
Schematic representation of the analysis of the percentage of selected subpopulations of T and B lymphocytes expressing positive CTLA-4 and CD86 among patients with CVID, CLL, and HV. (**A**) Percentage of CD4+ T cells positive for CTLA-4 expression; (**B**) percentage of CD8+ T cells positive for CTLA-4 expression; (**C**) percentage of CD19+ B cells positive for CTLA-4 expression; (**D**) percentage of CD4+ T cells positive for CD86expression; (**E**) percentage of CD8+ T cells positive for CD86 expression; and (**F**) percentage of CD19+ B cells positive for CD86 expression. * statistically significant results. Abbreviations: CVID—common variable immunodeficiency; CLL—chronic lymphocytic leukemia; CD—cell differentiation antigen; CTLA-4—cytotoxic T-lymphocyte-associated antigen 4; and HV—healthy volunteers.

**Figure 3:**
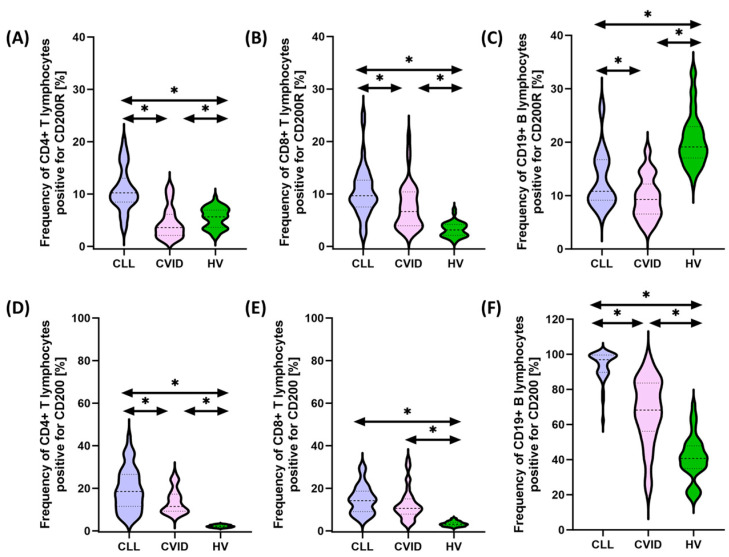
Schematic representation of the analysis of the percentage of selected subpopulations of T and B lymphocytes expressing positive CD200R and CD200 among patients with CVID, CLL, and HV. (**A**) Percentage of CD4+ T cells positive for CD200R expression; (**B**) percentage of CD8+ T cells positive for CD200R expression; (**C**) percentage of CD19+ B cells positive for CD200R expression; (**D**) percentage of CD4+ T cells positive for CD200 expression; (**E**) percentage of CD8+ T cells positive for CD200 expression; and (**F**) percentage of CD19+ B cells positive for CD200 expression. * statistically significant results. Abbreviations: CVID—common variable immunodeficiency; CLL—chronic lymphocytic leukemia; CD—cell differentiation antigen; and HV—healthy volunteers.

In recent years, interactions between hematological malignancies and immune checkpoints have received considerable attention in cancer research and treatment. Some malignant cells can use these checkpoints to evade immune surveillance, allowing them to proliferate and escape destruction by the immune system. This immune evasion mechanism has led to the development of new therapeutic approaches targeting immune checkpoints. Therefore, in the next stage of our research, we decided to compare the percentage of selected subpopulations of lymphocytes that showed positive expression of PD-1/PD-L1, CTLA-4/CD86, and CD200R/CD200 between the two tested populations of patients and healthy individuals. Detailed data obtained from this experiment are collected and presented in Supplementary Table S3 and Figure 1, Figure 2 and Figure 3.

It seems important to emphasize that all tested immunological checkpoints and their ligands showed higher expression both in patients with CVID and CLL in relation to healthy volunteers. In the case of the first group of patients, these changes were from 1.44 times (for CD8+ CD86+) to 25.78 times (for CD19+ PD-L1+) higher compared to the values recorded for control patients, while in the case of patients with SID, from 1.81 times (for CD4+CD200R+) to 30.30 times (for CD19+PD-L1+) higher (Supplementary Table S3 and Figure 1, Figure 2 and Figure 3).

Furthermore, we also noted several statistically significant changes between both subunits of the disease. These changes included an increase in the percentage of CD19+ PD-1+ (3.28-fold), CD8+ CTLA-4+ (1.36-fold), CD4+ CD200+ (1.61-fold), and CD86+ in all lymphocyte subpopulations tested (1.16-fold for CD4+, 1.33-fold for CD8+, and 1.57-fold for CD19+, respectively), and CD200 (1.61-fold for CD4+, 1.39-fold for CD8+, and 1.57-fold for CD19+, respectively) (42-fold for CD19+) and CD200R (2.85-fold for CD4+, 1.46-fold for CD8+, and 1.16-fold for CD19+, respectively) in CLL vs. CVID patients (Supplementary Table S3). No statistically significant differences were observed for PD-1 on CD4+ and CD8+ and PD-L1 on all subpopulations of T and B lymphocytes, as well as for CTLA-4 on CD4+ (Supplementary Table S3 and Figure 1, Figure 2 and Figure 3).

### 3.3. Analysis of Serum Concentrations of Selected Immunological Checkpoints and Their Ligands

**Figure 4:**
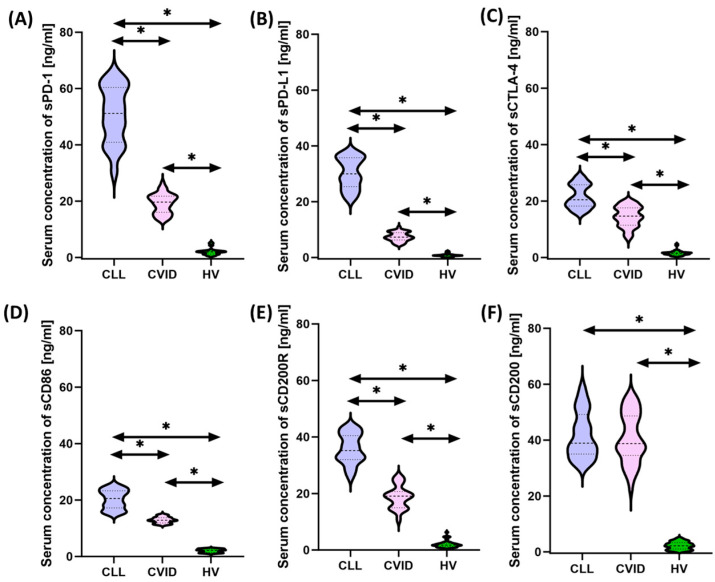
Schematic representation of the analysis of the serum concentration of soluble forms of the tested immune checkpoints and their ligands among patients with CVID, CLL, and HV. (**A**) Serum sPD-1 concentration; (**B**) serum PD-L1 concentrations; (**C**) serum sCTLA-4 concentration; (**D**) serum sCD86 concentration; (**E**) serum sCD200R concentration; and (**F**) serum sCD200 concentration; * statistically significant results. Abbreviations: CVID—common variable immunodeficiency; CLL—chronic lymphocytic leukemia; CD—cell differentiation antigen; PD-1—Programmed Cell Death Protein 1; PD-L1—Programmed Cell Death Ligand 1; CTLA-4—cytotoxic T-lymphocyte-associated antigen 4; and HV—healthy volunteers.

Due to several observed changes in the immunophenotype, in the next stage of the research, we decided to analyze whether the changes observed in the previous experiment will also be reflected in the assessment of the concentration of soluble from the tested immunological checkpoints and their ligands. The results obtained based on the performed immunoenzymatic tests are summarized in Supplementary Table S4 and Figure 4.

Statistical analysis of the obtained test results showed a significant increase in the concentration of soluble forms of all tested molecules in both CVID and CLL patients in relation to healthy volunteers, and the observed differences ranged from 5.81 times (for sCD86) to 17.86 times (for sCD200) for CVID patients and 14.11 fold (for CTLA-4) to 41.62 fold (for PD-L1) for CLL patients. Detailed differences between individual groups of patients from the study group showed higher values of all tested molecules among patients with CLL in relation to CVID, except sCD200.

### 3.4. Analysis of the Correlation of the Obtained Test Results with Biological Parameters

**Figure 5:**
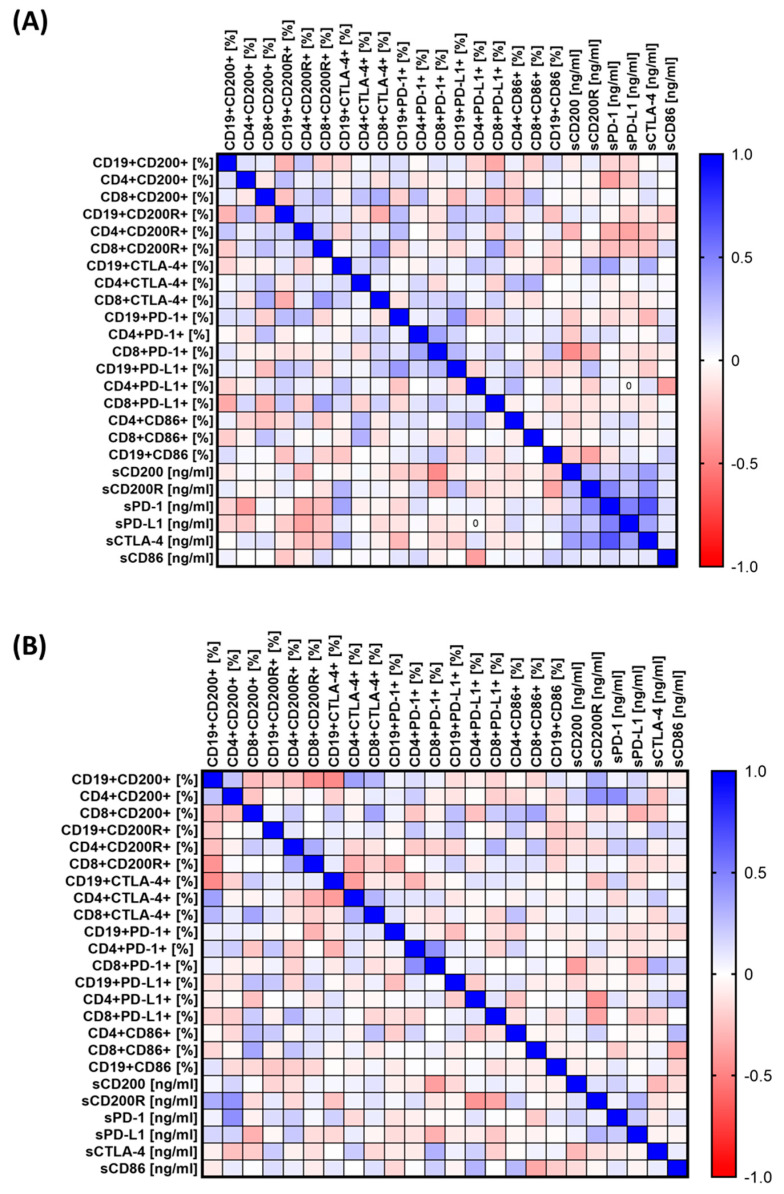
Schematic representation of the analysis of correlations of selected parameters of the functioning of the immune system of patients from the study group. (**A**) Correlations of immunological parameters in patients with CVID; (**B**) correlations of immunological parameters in CLL patients. Abbreviations: CVID—common variable immunodeficiency; CLL—chronic lymphocytic leukemia; CD—cell differentiation antigen; PD-1—Programmed Cell Death Protein 1; PD-L1—Programmed Cell Death Ligand 1; CTLA-4—cytotoxic T-lymphocyte-associated antigen 4; and HV—healthy volunteers.

Such varied changes observed between individual groups of patients caused us to assess whether there are any specific correlations between the assessed parameters of the immune system and their relationship with biological parameters. The results obtained are presented in Figure 5A and Supplementary Table S5 for patients with CVID and Figure 5B and Supplementary Table S6 for patients with CLL. In the case of patients with CVID, we recorded 40 statistically significant correlations, of which 13 were negative (nine low and four moderate) and the remaining 27 were positive (18 low, 9 moderate, and 1 high) (Supplementary Table S5). In the case of patients with CLL, 19 correlations were negative (7 moderate and 12 low), and the remaining 21 were positive correlations (7 moderate and 14 low) (Supplementary Table S6).

A correlation analysis of the tested immune system parameters with the number of infections in patients with CVID and CLL was also carried out. For patients with CVID, the analysis showed five statistically significant correlations (one negative and four positive) (Table 1). In the case of CLL patients, there are 11 statistically significant correlations (three negative and eight positive) (Table 1).

### 3.5. Assessment of the Potential Diagnostic Utility of Examined Immunological Checkpoints and Their Corresponding Ligands

**Figure 6:**
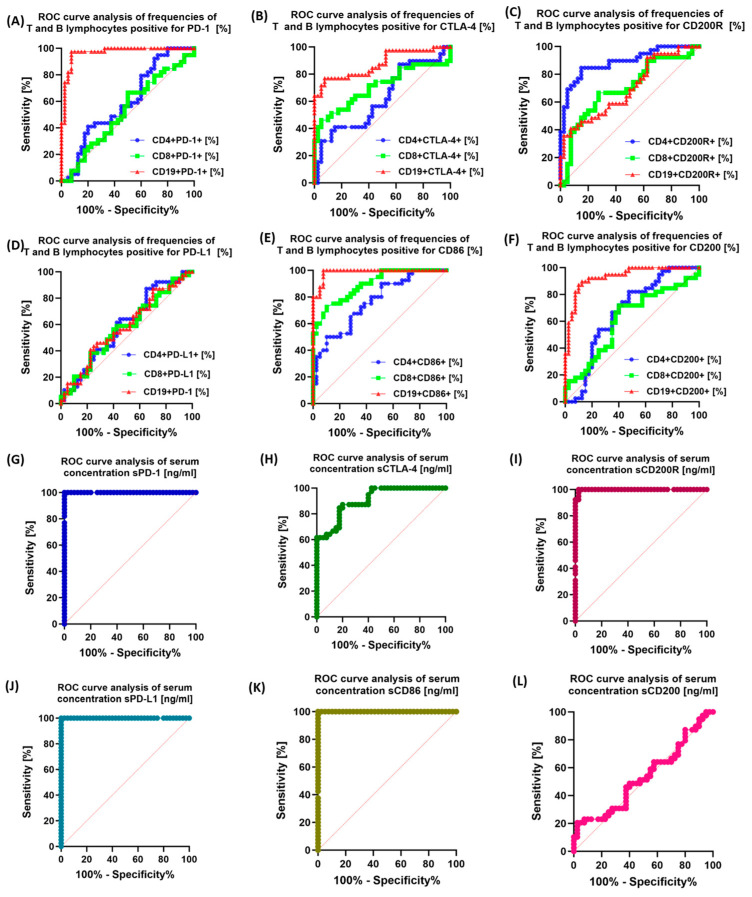
Analysis of ROC curves for the percentage of PD-1/PD-L1, CTLA-4/CD86, and CD200R/CD200 occurrence on selected T and B lymphocyte subpopulations and the raw concentration of tested molecules between the CLL and CVID patient groups. (**A**) ROC curve analysis of frequencies CD4+/CD8+ T lymphocytes and CD19+ B lymphocytes positive for PD-1 among CLL vs. CVID patients [%]; (**B**) ROC curve analysis of frequencies CD4+/CD8+ T lymphocytes and CD19+ B lymphocytes positive for CTLA-4 among CLL vs. CVID patients [%]; (**C**) ROC curve analysis of frequencies CD4+/CD8+ T lymphocytes and CD19+ B lymphocytes positive for CD200R among CLL vs. CVID patients [%]; (**D**) ROC curve analysis of frequencies CD4+/CD8+ T lymphocytes and CD19+ B lymphocytes positive for PD-L1 among CLL vs. CVID patients [%]; (**E**) ROC curve analysis of frequencies CD4+/CD8+ T lymphocytes and CD19+ B lymphocytes positive for CD86 among CLL vs. CVID patients [%]; (**F**) ROC curve analysis of frequencies CD4+/CD8+ T lymphocytes and CD19+ B lymphocytes positive for CD200 among CLL vs. CVID patients [%]; (**G**) ROC curve analysis of serum concentration of sPD-1 among CLL vs. CVID patients [ng/mL]; (**H**) ROC curve analysis of serum concentration of sCTLA-4 among CLL vs. CVID patients [ng/mL]; (**I**) ROC curve analysis of serum concentration of sCD200R among CLL vs. CVID patients [ng/mL]; (**J**) ROC curve analysis of serum concentration of sPD-L1 among CLL vs. CVID patients [ng/mL]; (**K**) ROC curve analysis of serum concentration of sCD86 among CLL vs. CVID patients [ng/mL]; and (**L**) ROC curve analysis of serum concentration of sCD200 among CLL vs. CVID patients [ng/mL].

The last stage of our research consisted of assessing the usefulness of selected immunological checkpoints and their ligands (both based on the immunophenotype and the concentration of soluble substances) as potential markers used in diagnostics. To this end, we performed ROC analyses of patients with CVID and CLL in relation to healthy volunteers, as well as analyzed their sensitivity between patients with CVID and CLL. The obtained test results are presented in Figure 6, Supplementary Figures S3–S5, and Supplementary Table S7. In the case of patients with CLL, compared to healthy volunteers, the most sensitive parameters turned out to be the percentage of PD-1 on all subpopulations of the tested lymphocytes and CD4+ CD200+, as well as the concentration of all tested molecules in the serum. For patients with CVID, the most sensitive parameters relative to controls may be the percentage of PD-1 and CD86 on all lymphocyte subpopulations tested, as well as CD8+ CTLA-4+ and CD4+ CD200+ and the serum concentration of all molecules tested.

The analysis of the use of the tested molecules in the context of their use as effective biomarker molecules showed the highest specificity of sPD-1 (Figure 6I) and sPD-L1 and sCD200R, and in the case of immunophenotypic analysis, also the percentage of CD19+ PD-1 and CD19+CD200+ (Figure 6, Supplementary Figures S3–S5, and Supplementary Table S7).

## 4. Discussion

SID occurring in individuals with B-cell hematologic malignancies is a prevalent state characterized by recurring infections. SID arises from both inherent immunological deficiencies caused by the malignancy itself and as a secondary effect of cancer therapies, many of which possess the capability to deplete B-cells. Prompt identification of SID and the enhancement of intervention approaches play a pivotal role in facilitating the most efficient cancer treatments and mitigating the incidence of infections and associated morbidity and mortality [7]. In the literature, we can find dozens of papers discussing in detail the disorders of the immune system in the course of SID, and in particular, CLL. In CLL, as with other cancers, the surveillance and response mechanisms of the immune system may be impaired. Immunological checkpoint assays are designed to assess the status of these checkpoints to better understand and potentially modulate the immune response against CLL cells [41]. PD-1 is a protein found on the surface of some immune cells, such as T cells. It interacts with PD-L1 and PD-L2, which are often overexpressed on cancer cells, including CLL cells. This interaction can suppress T cell activity, allowing cancer cells to evade detection by the immune system. Checkpoint inhibitors targeting the PD-1/PD-L1 pathway aim to block this interaction and enhance the immune response against cancer cells. This has been confirmed not only in our research but also in numerous studies by other scientists [42,43,44,45]. 

CTLA-4 is another checkpoint protein that regulates T-cell activation. It competes with the costimulatory molecule (CD28) for binding to antigen-presenting cells, suppressing T-cell responses. Inhibition of CTLA-4 may increase T-cell activity against tumor cells. As in the case of PD-1, it is one of the best-studied molecules in the course of CLL. The data presented in our publication are identical to the results of other researchers who also showed deregulation of CTLA-4 in the course of CLL (increased expression on selected populations of immune system cells) [46,47,48,49].

Much less research data can be found on the CD200R/CD200 pathway we studied in the course of CLL. CD200, an Ig-like type Ia transmembrane glycoprotein, is widely distributed in a variety of cell types, including B lymphocytes, a specific subset of T lymphocytes, dendritic cells, endothelial cells, and neurons. It transmits immunosuppressive signals through its receptor, CD200R, which is found on myeloid cells and T lymphocytes. In addition, the interaction of CD200 with CD200R is involved in the regulation of tumor immunity. While CLL and hairy cell leukemia show elevated expression of CD200, mantle cell lymphoma lacks this marker, allowing better differentiation between these different B-cell malignancies, each of which has a different prognosis [50,51]. In most studies available in the literature, as well as in our own, CD200 and CD200R expression is upregulated on immune cells in CLL [50,51,52]. According to the scientists, however, further intensive research is needed, which in the future may open the way to a new targeted therapy (anti-CD200).

While in the literature we can find many important reports on the study of immunological checkpoints in the course of CLL, the information on their significance in the course of PID, and in particular CVID, and their relationship with the development of hematological malignancies is quite limited. The most information on the development of hematological malignancies in patients with CVID is provided by the analysis by Kiaee et al. from 2018 [29]. This team conducted a detailed analysis of 48 literature studies covering a total of 8123 CVID patients with cancer. Researchers indicated that the incidence of malignant tumors was 8.6%, of which lymphoma was the most common, at 4.1%, gastric cancer was at 1.5%, and breast cancer was at 1.3%. The researchers also point out that their meta-analysis suggests that autoimmunity and malabsorption were more common in cancer patients than in non-cancer patients. In their publication, the authors pay special attention to the fact that the incidence of malignant tumors in patients with CVID has increased due to improved survival rates, but the importance of expanding and implementing screening tests as well as developing strategies for the treatment of malignant tumors in patients with CVID is important [29]. 

Other research by the Bruns team in 2022 [25] indicates that the extent of cell-mediated immunodeficiency in CVID appears to be related to the diagnosis of cancer, which is consistent with the concept of immune surveillance against cancer.

This is confirmed by research by Brent et al. A 2016 cohort of 801 people with primary hypogammaglobulinemia, including CVID, revealed that cancer patients had reduced CD8+ T cell counts. In addition, patients with non-hematologic malignancies had significantly lower B-cell counts [53]. Also, in the results of the Kiaee team, people with CVID with cancer showed a trend towards a decrease in the percentage of CD8+ T cells [29]. Research by Lougaris et al. in 2015 [54], Wehr in 2016 [55], and Wong and Huissoon from 2016 [56] indicate that the percentage of CD8+ T cells showed no significant discrepancy, and similar percentages of other lymphocyte subsets tested were observed between CVID patients with and without CVID cancer. Evaluation of immunological parameters, including B cells, NK cells, and CD8+ T cells during CVID diagnosis, revealed no discernible differences between cancer and non-cancer patients. Still, the lack of significant changes in NK or CD8+ T cells seems to challenge the concept of tumor immune surveillance. Nevertheless, cases of T cell or NK cell dysfunction have been reported in patients with CVID, which may be relevant to the development of CVID-associated malignancies. 

In CVID, the intricate balance of the immune system is disrupted, leading to inappropriate immune responses and chronic inflammation. This disruption may involve checkpoint molecules that play a role in regulating immune activation and tolerance. Although the exact mechanisms remain to be fully elucidated, examining immune checkpoints in CVID can provide insight into underlying immune system dysfunctions. However, the literature data on this issue are extremely poor. Few literature data emphasize the significantly higher expression of PD-1 and CTLA-4 on selected populations of immune system cells in the course of CVID compared to healthy volunteers, which is consistent with the results of our study [56,57,58,59,60]. The causes of malignant transformation in CVID are still being intensively investigated, but researchers suggest that it is largely due to impaired immune surveillance. The consequences of this are the occurrence of secondary mutations and defective mechanisms of identification and elimination of replaced cancer cells, as well as infections with oncogenic viruses, as well as long-term stimulation of the immune system.

Despite the significance of these concerns, there is a notable absence of comprehensive studies in the existing literature that examine a wide array of immunological checkpoints and their corresponding ligands concerning PID or SID, particularly in relation to CVID and CLL. Consequently, research findings presented in this publication represent one of the initial comprehensive endeavors to elucidate the immune system dysregulation through immune checkpoints in the progression of immunodeficiencies. We hope that the presented small part of the changes in the functioning of the immune system in patients with CVID or CLL will help to deepen the knowledge about disorders of immune homeostasis and will inspire further detailed research on the role of immune checkpoints, which in the future may be used to develop new diagnostic and therapeutic strategies for all patients with immunodeficiencies, as well as to monitor disease progression, which will reduce the risk of cancer, including hematological cancer.

## 5. Conclusions

It is important to remember that although some immunodeficiency disorders may increase the likelihood of hematological malignancies, not all immunodeficient individuals will develop cancer. Similarly, not all people with hematologic malignancies will develop immunodeficiency. Nevertheless, this balance is fragile, and any disturbance in the functioning of the immune system can trigger harmful mechanisms that promote the formation of cancer. Therefore, continuous monitoring of the immune system in immunodeficiency patients becomes of utmost importance. Our research, while part of a broader spectrum of changes seen in CVID and CLL, shows promise for clinicians, oncologists, and patients, highlighting the importance of being vigilant about immune checkpoint dysregulation. Currently, the relationship between CVID and malignancies is not considered in standard clinical practice, resulting in a lack of routine cancer screening for prevention and early detection in patients with CVID.

While checkpoint inhibitors have shown remarkable effectiveness in specific cases, they do not always benefit all patients or types of hematological cancers. Additionally, immunotherapy may lead to immune-related side effects, requiring careful monitoring and treatment. The presented results of studies on newly diagnosed patients are a starting point for further detailed and interdisciplinary research on the role of immune checkpoints in the context of immunodeficiencies and hematological malignancies. These studies, along with other immunotherapy approaches, may play an increasingly key role in the treatment and monitoring of these disease groups in the future.

## Figures and Tables

**Table 1:** Correlation analysis of the tested parameters of the immune system and number of infections requiring antibiotic therapy in the last 12 months before diagnosis in patients with CVID and CLL.

**Correlations of Immunological Parameters with the Number of Infections for CVID Patients**			
**A Pair of Variables**	**R**	**t (*n* − 2)**	***p***
CD8+ CD200+ and gastrointestinal infections	−0.331	−2132	0.040 *
CD4+ CTLA-4+ and skin infections	0.365	2386	0.022 *
CD8+ PDL-1+ and gastrointestinal infections	0.483	3357	0.002 *
CD8+ PDL-1+ and total number of infections	0.458	3136	0.003 *
sPD-L1 and skin infections	0.318	2042	0.048 *
**Correlations of Immunological Parameters with the Number of Infections for CLL Patients**			
**A Pair of Variables**	**R**	**t (*n* − 2)**	***p***
CD4+ CD200+ and infections of the upper and lower respiratory tract	0.374	2484	0.018 *
CD4+ CD200+ and Skin infections	0.380	2534	0.016 *
CD4+ CD200+ and total number of infections	0.477	3346	0.002 *
CD19+ CD200R+ and gastrointestinal infections	0.349	2294	0.027 *
CD4+ CD200R+ and urinary tract infections	−0.344	−2260	0.030 *
CD8+ CD200R+ and gastrointestinal infections	0.325	2118	0.041 *
CD8+ CTLA-4+ and urinary tract infections	0.376	2502	0.017 *
CD8+ PD-1+ and infections of the upper and lower respiratory tract	0.315	2046	0.048 *
CD8+ PDL-1+ and infections of the upper and lower respiratory tract	−0.366	−2425	0.020 *
CD8+ PDL-1+ and total number of infections	−0.394	−2646	0.012 *
sPD-L1 and skin infections	0.349	2293	0.027 *

* Statistically significant results

## Data Availability

All necessary information regarding the preparation of this work is available on written request from the corresponding author.

## Supplementary Materials

The following supporting information can be downloaded at https://www.mdpi.com/article/10.3390/cancers15215184/s1, Figure S1: Sample flow cytometry analysis and a gating strategy to evaluate subpopulations of T and B lymphocytes; Figure S2: Schematic representation of the number of infections requiring antibiotic therapy in CVID and CLL patients and healthy volunteers during the last 12 months after diagnosis; Figure S3: Analysis of ROC curves for the percentage of PD-1/PD-L1 occurrence on selected T and B lymphocyte subpopulations and the concentration of tested molecules between the tested patient groups; Figure S4: Analysis of ROC curves for the percentage of CTLA-4/CD86 occurrence on selected T and B lymphocyte subpopulations and the concentration of tested molecules between the tested patient groups; Figure S5: Analysis of ROC curves for the percentage of CD200R/CD200 occurrence on selected T and B lymphocyte subpopulations and the concentration of tested molecules between the tested patient groups; Table S1: Selected parameters of morphology and biochemistry of peripheral blood; Table S2: The peripheral blood immunophenotype of patients with CVID and CLL in comparison to that of HV; Table S3: Analysis of the percentage of selected subpopulations of peripheral blood lymphocytes showing positive expression of the tested immunological checkpoints and their ligands; Table S4: The concentration of soluble forms of the tested immunological checkpoints and their ligands in the serum of immunodeficient patients about healthy volunteers; Table S5: Correlation analysis of the tested parameters of the immune system and peripheral blood morphology and biochemistry in patients with CVID; Table S6: Correlation analysis of the tested parameters of the immune system and peripheral blood morphology and biochemistry in patients with CLL; Table S7: ROC curve analysis.
