# Supplementary material for: Immune Checkpoint Pathway Expression in Lymphocyte Subpopulations in Patients with Common Variable Immunodeficiency and Chronic Lymphocytic Leukemia
Source: Cancers (Basel). 2023 Oct 28;15(21):5184. doi: 10.3390/cancers15215184 (PMC10649987; doi:10.3390/cancers15215184)
Supplement: Supplementary file 1 [file cancers-15-05184-s001.zip › cancers-2572221-supplementary.pdf]

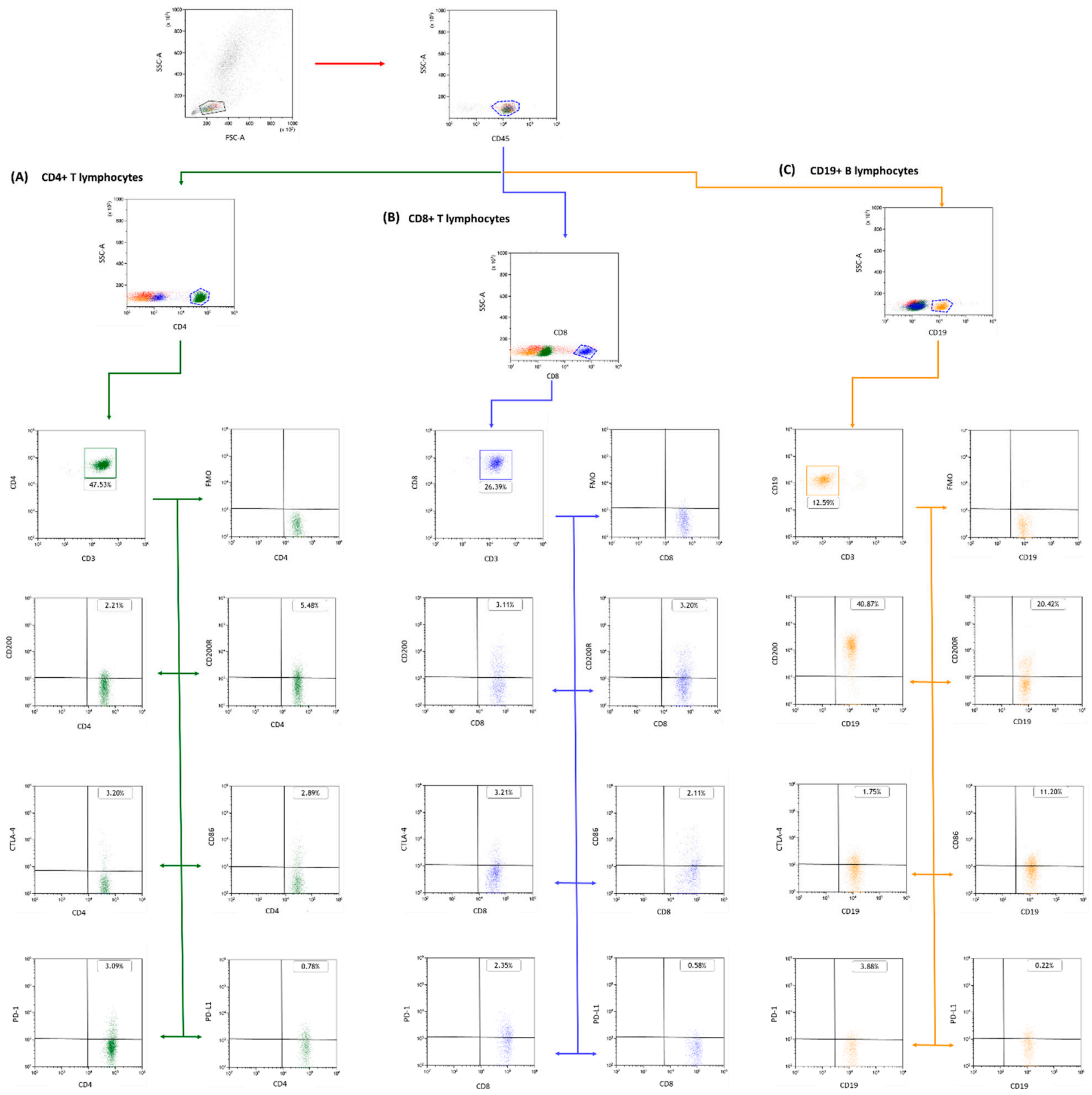

Supplementary Figure S1. Sample flow cytometry analysis and a gating strategy to evaluate subpopulations of T and B lymphocytes. We measured the percentage of positive events for CD4+CD3+ (green), CD8+CD3+ (blue), and CD19+CD3- (orange) cells, against molecules such as CD200, CD200R, CTLA-4, CD86, PD-1, and PD-L1. To ensure the accuracy of our findings, we included Fluorescence Minus One (FMO) controls as negative controls to establish the appropriate gating thresholds. The example analysis concerns a patient from the HV group.

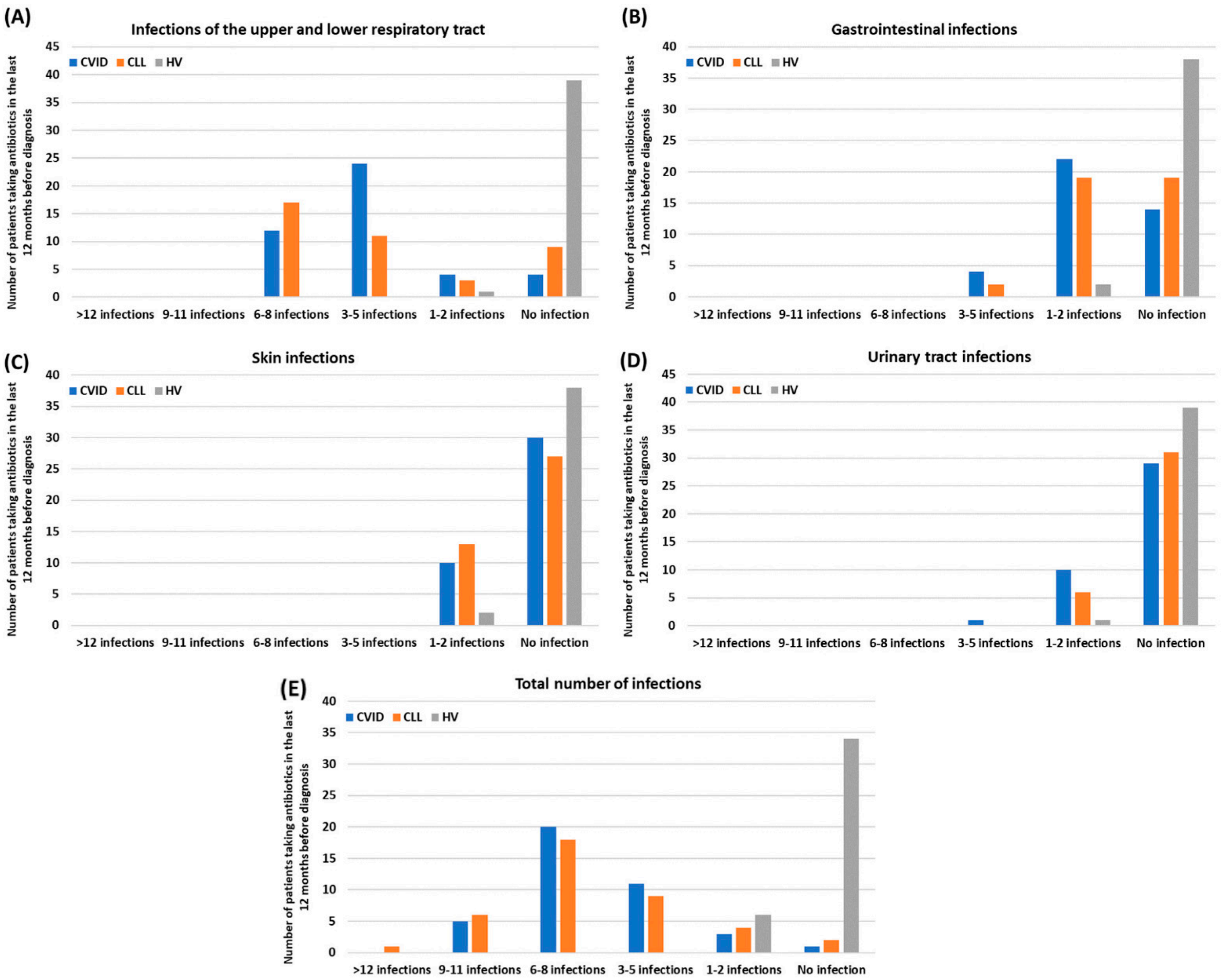

Supplementary Figure S2. Schematic representation of the number of infections requiring antibiotic therapy in CVID and CLL patients and healthy volunteers during the last 12 months after diagnosis. (A) Number of upper and lower respiratory tract infections requiring antibiotic therapy in the last 12 months; (B) Number of gastrointestinal infections requiring antibiotic therapy in the past 12 months; (C) Number of skin infections requiring antibiotic therapy in the past 12 months; (D) Number of urinary tract infections requiring antibiotic therapy in the past 12 months; (E) total number of all infections requiring antibiotic therapy in the last 12 months.

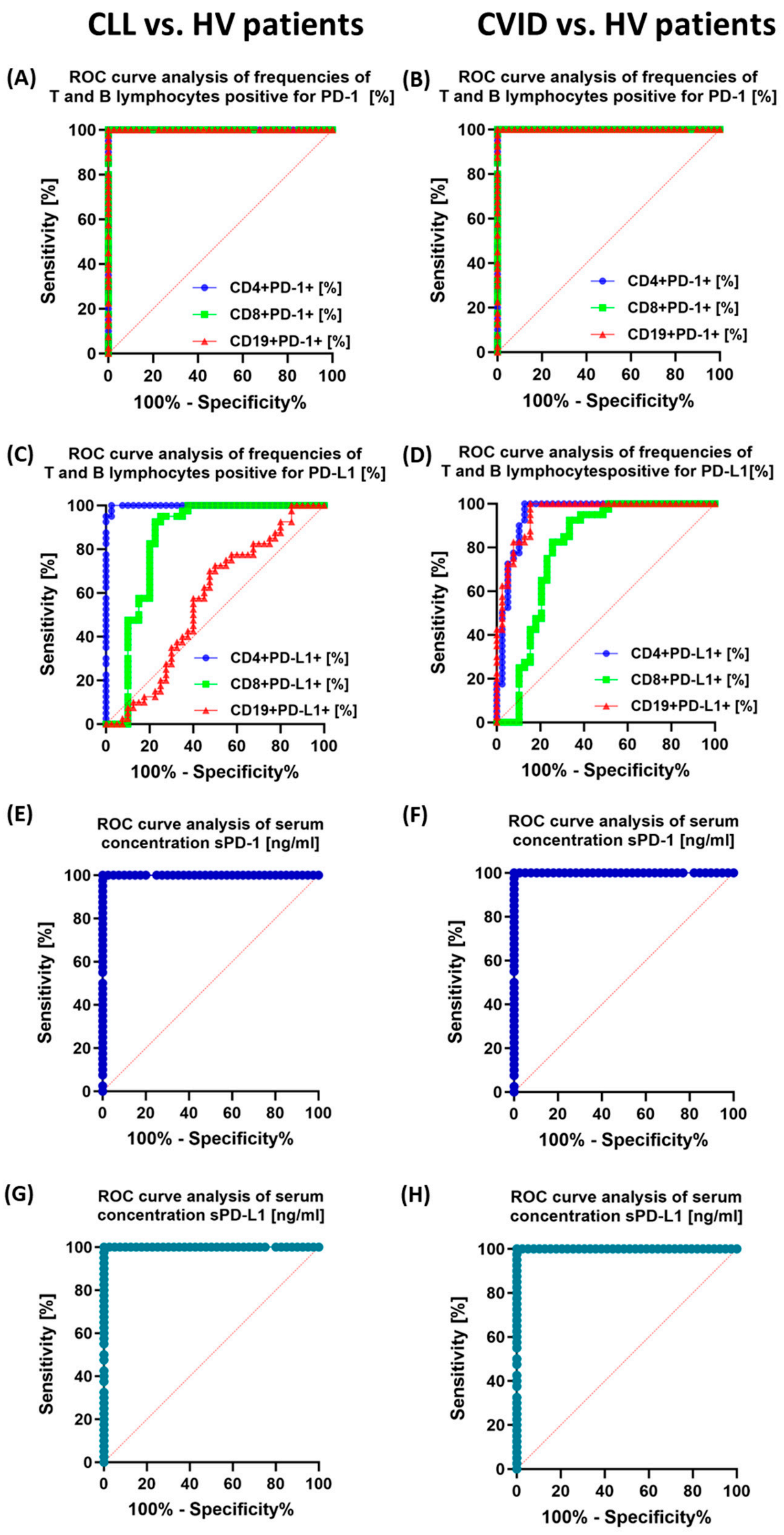

Supplementary Figure S3 Analysis of ROC curves for the percentage of PD-1/PD-L1 occurrence on selected T and B lymphocyte subpopulations and the raw concentration of tested molecules between the tested patient groups. (A) ROC curve analysis of frequencies CD4+/CD8+ T lymphocytes and CD19+ B lymphocytes positive for PD-1 among CLL vs. HV patients [%];(B) ROC curve analysis of frequencies CD4+/CD8+ T lymphocytes and CD19+ B lymphocytes positive for PD-1 among CVID vs. HV patients [%]; (C) ROC curve analysis of frequencies CD4+/CD8+ T lymphocytes and CD19+ B lymphocytes positive for PD-L1 among CLL vs. HV patients [%];(D) ROC curve analysis of frequencies CD4+/CD8+ T lymphocytes and CD19+ B lymphocytes positive for PD-L1 among CVID vs. HV patients [%]; (E) ROC curve analysis of serum concentration sPD-1 among CLL vs. HV patients [ng/ml]; (F) ROC curve analysis of serum concentration sPD-1 among CVID vs. HV patients [ng/ml]; (G) ROC curve analysis of serum concentration sPD-L1 among CLL vs. HV patients [ng/ml]; (H) ROC curve analysis of serum concentration sPD-L1 among CVID vs. HV patients [ng/ml].

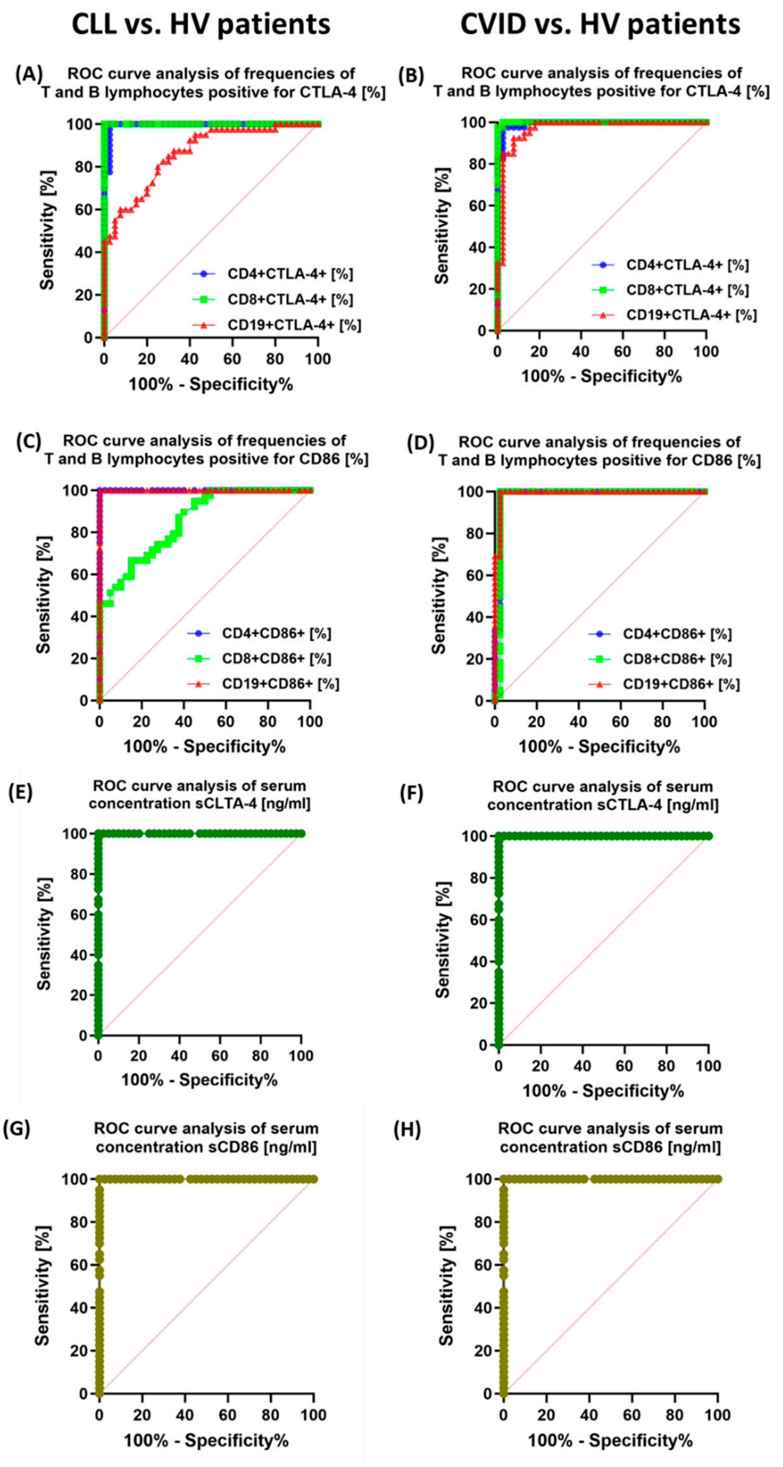

Supplementary Figure S4. Analysis of ROC curves for the percentage of CTLA-4/CD86 occurrence on selected T and B lymphocyte subpopulations and the raw concentration of tested molecules between the tested patient groups. (A) ROC curve analysis of frequencies CD4+/CD8+ T lymphocytes and CD19+ B lymphocytes positive for CTLA-4among CLL vs. HV patients [%];(B) ROC curve analysis of frequencies CD4+/CD8+ T lymphocytes and CD19+ B lymphocytes positive for CTLA-4 among CVID vs. HV patients [%]; (C) ROC curve analysis of frequencies CD4+/CD8+ T lymphocytes and CD19+ B lymphocytes positive for CD86 among CLL vs. HV patients [%];(D) ROC curve analysis of frequencies CD4+/CD8+ T lymphocytes and CD19+ B lymphocytes positive for CD86 among CVID vs. HV patients [%]; (E) ROC curve analysis of serum concentration sCTLA-4 among CLL vs. HV patients [ng/ml]; (F) ROC curve analysis of serum concentration sCTLA-4 among CVID vs. HV patients [ng/ml]; (G) ROC curve analysis of serum concentration sCD86 among CLL vs. HV patients [ng/ml]; (H) ROC curve analysis of serum concentration sCD86 among CVID vs. HV patients [ng/ml].

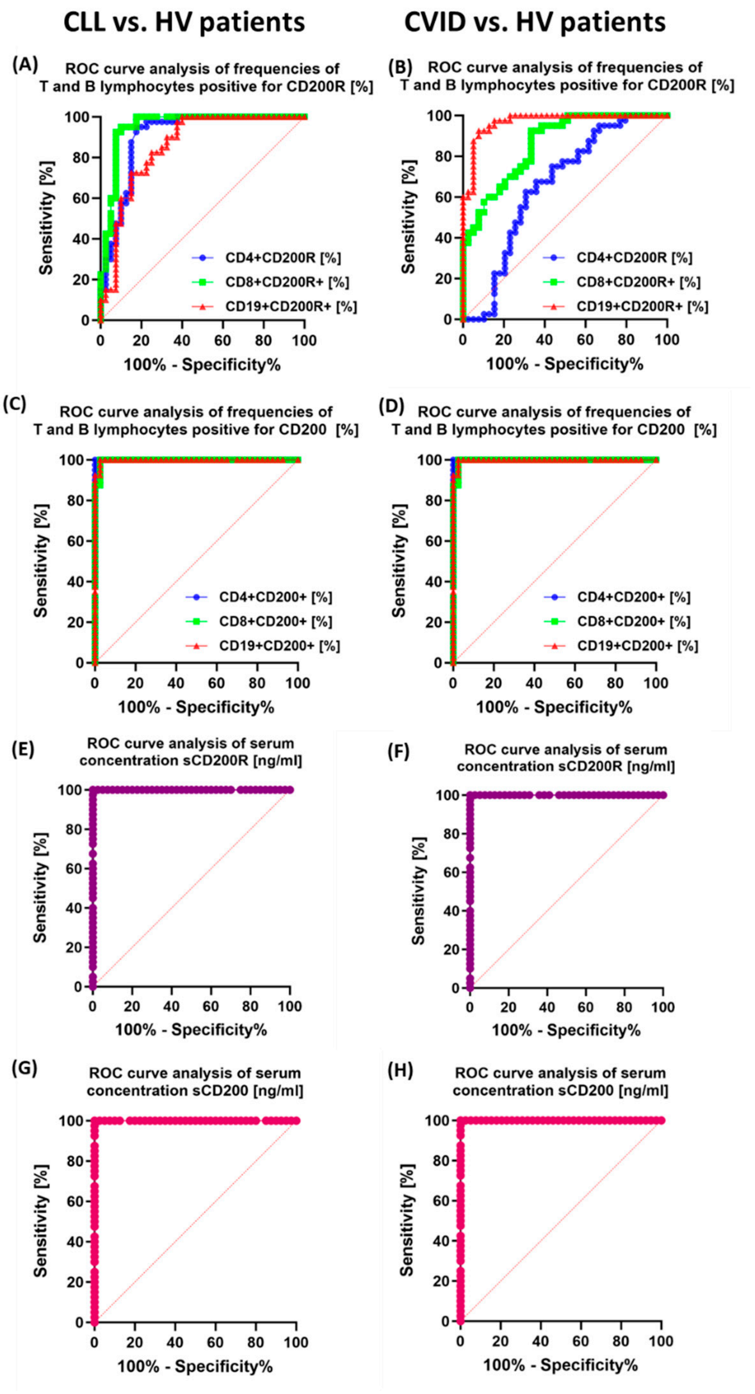

Supplementary Figure S5. Analysis of ROC curves for the percentage of CD200R/CD200 occurrence on selected T and B lymphocyte subpopulations and the raw concentration of tested molecules between the tested patient groups. (A) ROC curve analysis of frequencies CD4+/CD8+ T lymphocytes and CD19+ B lymphocytes positive for CD200R among CLL vs. HV patients [%];(B) ROC curve analysis of frequencies CD4+/CD8+ T lymphocytes and CD19+ B lymphocytes positive for CD200R among CVID vs. HV patients [%]; (C) ROC curve analysis of frequencies CD4+/CD8+ T lymphocytes and CD19+ B lymphocytes positive for CD200 among CLL vs. HV patients [%];(D) ROC curve analysis of frequencies CD4+/CD8+ T lymphocytes and CD19+ B lymphocytes positive for CD200 among CVID vs. HV patients [%]; (E) ROC curve analysis of serum concentration sCD200R among CLL vs. HV patients [ng/ml]; (F) ROC curve analysis of serum concentration sCD200R among CVID vs. HV patients [ng/ml]; (G) ROC curve analysis of serum concentration sCD200 among CLL vs. HV patients [ng/ml]; (H) ROC curve analysis of serum concentration sCD200 among CVID vs. HV patients [ng/ml].

Supplementary Table S1. Selected parameters of morphology and biochemistry of peripheral blood

Parameter	CVID (n = 40)		CLL (*n* = 40)		HV (*n* = 40)		*p*-value	*p*-value		
	Mean ± SD	Median (Range)	Mean ± SD	Median (Range)	Mean ± SD	Median (Range)		CVID vs. CLL	CVID vs. HV	CLL vs. HV
**WBC**	6.92 ± 1.63	6.7 (4.78-9.99)	31.95± 11.35	34.19 (10.34-47.85)	5.66 ± 1.39	6.03 (3.40-7.78)	0.000*	0.000*	0.000*	0.005*
**LYM**	0.96± 0.54	0.92 (0.08-1.99)	12.16± 5.21	11.26 (5.54- 35.60)	1.56 ± 0.49	1.49 (0.76-2.85)	0.000*	0.000*	0.000*	0.000*
**MON**	0.47± 0.15	0.47 (0.20-0.79)	0.57± 0.45	0.42 (0.09-1.71)	0.48± 0.15	0.48 (0.20-0.81)	0.8394	0.601	0.524	0.629
**NEU**	1.03± 0.56	1.14 (0.03-1.92)	3.31± 1.44	3.15 (0.61-7.40)	3.35± 1.27	3.50 (0.91-5.52)	0.000*	0.000*	0.6288	0.000*
**EOS**	0.08± 0.03	0.08 (0.03-0.19)	0.12± 0.10	0.10 (0.01-0.51)	0.10± 0.04	0.09 (0.03-0.22)	0.1510	0.169	0.698	0.047
**BAS**	0.03± 0.02	0.03 (0.00-0.08)	0.08± 0.05	0.07 (0.01-0.21)	0.04± 0.02	0.03 (0.00-0.09)	0.000*	0.000*	0.000*	0.1136
**RBC**	3.39± 0.24	3.39 (2.86-3.89)	4.21± 0.70	4.18 (2.34-5.21)	4.51± 0.32	4.53 (3.81-5.19)	0.000*	0.000*	0.038*	0.000*
**HGB**	9.98± 0.67	9.98 (8.33-11.63)	12.27± 1.71	12.50 (8.20-14.40)	13.30± 0.90	13.30 (11.10-15.50)	0.000*	0.000*	0.0044*	0.000*
**PLT**	115.43± 18.24	114.09 (86.36-148,42)	122.85± 41.52	116.00 (34.00-198.00)	244,50± 64.11	233.50 (152-404)	0.000*	0.3459	0.000*	0.000*
**IgG**	5.08± 0.86	5.19 (2.96-6.58)	5.37± 3.51	5.32 (0.31-12.93)	12.40± 2.11	12.65 (7.23-16.06)	0.000*	0.7661	0.000*	0.000*
**IgM**	1.37± 0.53	1.38 (0.37-2.38)	0.58± 0.79	0.32 (0.05-3.38)	1.47± 0.57	1.49 (0.40-2.52)	0.000*	0.000*	0.000*	0.392
**IgA**	0.62± 0.23	0.64 (0.11-1.09)	1.79± 1.10	1.44 (0.23-4.37)	2.07± 0.76	1.95 (0.70-4.00)	0.000*	0.000*	0.048	0.000*

* Statistically significant results; Abbreviations: BAS- basophil; EOS- eosinophil; HGB- hemoglobin; IgA- immunoglobulin A; IgG- immunoglobulin G; IgM- immunoglobulin M; LYM- lymphocyte; MON- monocyte; NEU-neutrophil; PLT- platelet; RBC- red blood cell; WBC-white blood cell.

Supplementary Table S2. The peripheral blood immunophenotype of patients with CVID and CLL in comparison to that of HV.

Parameter	CVID (*n* = 40)		CLL (*n* = 40)		HV (*n* = 40)		*p*-value	*p*-value		
	Mean ± SD	Median (Range)	Mean ± SD	Median (Range)	Mean ± SD	Median (Range)		CVID vs. CLL	CVID vs. HV	CLL vs. HV
**Frequencies of CD45+ leukocytes [%]**	92.88± 5.76	92.07 (88.75-98.79)	96.05± 8.28	95.50 (84.83-98.42)	97.28± 2.75	97.88 (89.14-99.37)	0.000*	0.000*	0.000*	0.000*
**Frequencies of CD3+ T lymphocytes [%]**	67.23± 9.66	66.40 (44.85-89.85)	14.20± 7.54	12.24 (3.47-33.41)	74.83± 6.31	71.94 (65.09-96.34)	0.000*	0.000*	0.000*	0.003*
**Frequencies of CD19+ B lymphocytes [%]**	9.74± 5.37	8.71 (2.81-24.17)	82.01± 10.54	83.52 (53.66-97.02)	12.59± 2.25	12.46 (7.91-16.83)	0.000*	0.000*	0.000*	0.000*
**Frequencies of CD4+CD3+ T lymphocytes [%]**	31.30± 12.22	28.98 (14.49-67.23)	6.43± 3.23	5.92 (1.52-26.57)	47.53± 4.76	46.76 (42.19-65.51)	0.000*	0.000*	0.000*	0.000*
**Frequencies of CD8+CD3+ T lymphocytes [%]**	31.30±13.23	28.13 (12.25-57.20)	5.03±3.16	4.26 (0.58-13.08)	26.39± 2.98	26.91 (20.18-31.07)	0.000*	0.000*	0.000*	0.387
**CD4+CD3+/CD8+CD3+ lymphocyte ratio**	1.49± 1.50	1.07 (0.26-3.16)	1.43± 0.74	1.28 (0.34-3.95)	1.80±1.39	1.74 (1.09-3.19)	0.000*	0.000*	0.000*	0.001*

* Statistically significant results; Abbreviations: CD- cluster of differentiation

Supplementary Table S3. Analysis of the percentage of selected subpopulations of peripheral blood lymphocytes showing positive expression of the tested immunological checkpoints and their ligands

Parameter		CVID (*n* = 40)		CLL (*n* = 40)		HV (*n* = 40)		*p*-value	*p*-value		
		Mean ± SD	Median (Range)	Mean ± SD	Median (Range)	Mean ± SD	Median (Range)		CVID vs. CLL	CVID vs. HV	CLL vs. HV
**PD-1**	**CD4+PD-1+ T lymphocytes [%]**	14.36± 4.70	14.41 (6.52-24.72)	18.34± 9.84	14.85 (4.55-44.00)	3.09± 1.14	3.00 (1.04-5.68)	0.000*	0.657	0.000*	0.000*
	**CD8+PD-1+ T lymphocytes [%]**	14.58± 7.20	13.39 (5.17-37.47)	14.72± 6.49	14.57 (3.27-29.16)	2.35± 0.98	2.26 (0.79-4.28)	0.000*	0.135	0.000*	0.000*
	**CD19+PD-1+ B lymphocytes [%]**	6.07± 2.98	6.01 (2.01-17.59)	19.86± 6.78	19.72 (5.33-35.65)	3.88± 1.38	3.73 (1.46-6.82)	0.000*	0.000*	0.000*	0.000*
**PD-L1**	**CD4+PD-L1+ T lymphocytes [%]**	7.80± 2.79	7.86 (2.55-13.92)	8.91± 3.30	8.99 (2.96-16.15)	0.78± 0.30	0.81 (0.21-1.33)	0.000*	0.166	0.000*	0.000*
	**CD8+PD-L1+ T lymphocytes [%]**	5.99± 3.49	5.09 (1.06-17.00)	6.90± 4.01	5.86 (1.23-19.72)	0.58± 0.25	0.57 (0.01-1.01)	0.000*	0.284	0.000*	0.000*
	**CD19+PD-L1+ B lymphocytes [%]**	7.21± 4.13	5.93 (1.62-17.87)	8.47± 4.77	6.97 (1.88-20.73)	0.22± 0.14	0.23 (0.00-0.48)	0.000*	0.201	0.000*	0.000*
**CTLA-4**	**CD4+CTLA-4+ T lymphocytes [%]**	10.34± 4.75	9.60 (4.17-23.55)	12.35± 5.54	10.33 (4.03-31.14)	3.20± 0.68	3.23 (1.91-4.41)	0.000*	0.0657	0.000*	0.000*
	**CD8+CTLA-4+ T lymphocytes [%]**	13.12± 7.86	11.28 (4.16-36.89)	15.91± 3.91	15.35 (8.36-26.69)	3.21± 0.82	3.01 (2.09-5.12)	0.000*	0.0014*	0.000*	0.000*
	**CD19+CTLA-4+ B lymphocytes [%]**	5.92± 2.66	6.22 (1.15-10.89)	13.58± 6.20	13.57 (3.12-29.42)	1.75± 0.65	1.75 (0.59-3.17)	0.000*	0.000*	0.000*	0.000*
**CD86**	**CD4+CD86+ T lymphocytes [%]**	4.84± 0.58	4.69 (4.02-5.99)	5.53±0.79	5.44 (4.16-6.97)	2.89± 0.62	2.80 (2.04-3.98)	0.000*	0.000*	0.000*	0.000*
	**CD8+CD86+ T lymphocytes [%]**	2.96± 0.59	2.95 (2.03-3.98)	4.00± 0.56	3.92 (3.01-4.95)	2.11± 0.51	2.05 (1.17-2.99)	0.000*	0.000*	0.000*	0.000*
	**CD19+CD86+ B lymphocytes [%]**	25.21±1.89	25.15 (22.18-28.00)	39.20±6.51	39.61 (27.24-48.52)	11.20± 1.84	11.61 (8.27-13.97)	0.000*	0.000*	0.000*	0.000*
**CD200R**	**CD4+CD200R+ T lymphocytes [%]**	4.50± 2.97	3.59 (1.13-11.52)	10.84± 4.10	10.23 (3.23-20.39)	5.48± 1.90	5.64 (1.89-9.31)	0.000*	0.000*	0.000*	0.016*
	**CD8+CD200R+ T lymphocytes [%]**	7.64± 4.330	6.63 (2.46-21.02)	10.51± 4.76	9.66 (2.09-25.33)	3.20± 1.36	3.15 (0.74-7.10)	0.000*	0.0022*	0.000*	0.000*
	**CD19+CD200R+ B lymphocytes [%]**	9.58± 3.88	9.27 (3.51-18.41)	13.05± 5.28	10.80 (6.18-27.33)	20.42± 4.82	19.11 (12.55-33.00)	0.000*	0.0022*	0.000*	0.000*
**CD200**	**CD4+CD200+ T lymphocytes [%]**	13.72± 5.69	11.47 (6.36-27.10)	19.39± 9.87	18.50 (5.32-43.59)	2.21± 0.57	2.15 (1.20-3.30)	0.000*	0.0103*	0.000*	0.000*
	**CD8+/CD200+ T lymphocytes [%]**	12.50± 7.41	10.55 (3.01-33.61)	14.46± 6.58	14.15 (4.59-30.69)	3.11± 1.07	3.14 (1.40-5.81)	0.000*	0.008*	0.000*	0.000*
	**CD19+/CD200+ B lymphocytes [%]**	67.22± 18.68	68.25 (23.01-96.27)	93.69± 8.00	96.91 (62.39-99.99)	40.87± 12.59	40.68 (18.15-71.33)	0.000*	0.000*	0.000*	0.000*

* Statistically significant results; Abbreviations: CVID - common variable immunodeficiency; CLL- chronic lymphocytic leukemia; CD- cell differentiation antigen; CTLA-4- cytotoxic T-lymphocyte–associated antigen 4; PD-1-Programmed Cell Death Protein 1; PD-L1 - Programmed Cell Death Ligand 1; HV- healthy volunteers.

Supplementary Table S4. The concentration of soluble forms of the tested immunological checkpoints and their ligands in the serum of immunodeficient patients about healthy volunteers.

Serum concentration (ng/ml)	CVID (*n* = 40)		CLL (*n* = 40)		HV (*n* = 40)		*p*-value	*p*-value		
	Mean ± SD	Median (Range)	Mean ± SD	Median (Range)	Mean ± SD	Median (Range)		CVID vs. CLL	CVID vs. HV	CLL vs. HV
**sPD-1**	19.20± 3.48	19.66 (13.19-26.49)	50.57± 9.91	51.20 (30.17-64.69)	2.10± 1.19	1.98 (0.52-5.42)	0.000*	0.000*	0.000*	0.000*
**sPD-L1**	7.44± 1.61	7.31 (4.46-10.89)	30.22± 5.45	29.97 (20.50-37.97)	0.77± 0.50	0.72 (0.04-2.25)	0.000*	0.000*	0.000*	0.000*
**sCTLA-4**	14.50± 3.56	14.66 (6.75-19.96)	21.60± 4.06	20.46 (15.56-28.84)	1.54± 0,88	1.45 (0.40-4.74)	0.000*	0.000*	0.000*	0.000*
**sCD86**	12.92± 1.06	12.85 (11.23-14.95)	20.12±3.38	20.39 (15.03-25.42)	2.05± 0.62	2.21 (1.03-2.95)	0.000*	0.000*	0.000*	0.000*
**sCD200R**	18.77± 3.96	19.09 (10.37-26.22)	35.85± 5.22	35.20 (25.39-43.96)	2.09± 1.19	1.76 (0.96-6.29)	0.000*	0.000*	0.000*	0.000*
**sCD200**	40.73± 8.82	38.75 (22.79-55.46)	41.79± 8.13	38.90 (30.65-59.25)	2.22± 1.34	2.17 (0.11-4.87)	0.000*	0.622	0.000*	0.000*

* Statistically significant results; Abbreviations: CVID - common variable immunodeficiency; CLL- chronic lymphocytic leukemia; CD- cell differentiation antigen; sCD86- soluble form of CD86; sCD200R- soluble form of CD200R; sCD200- soluble form of CD200; sCTLA-4- soluble form of cytotoxic T-lymphocyte–associated antigen 4; sPD-1- soluble form of Programmed Cell Death Protein 1; sPD-L1 - soluble form of Programmed Cell Death Ligand 1; HV- healthy volunteers.

Supplementary Table S5. Correlation analysis of the tested parameters of the immune system and peripheral blood morphology and biochemistry in patients with CVID

A pair of variables	R Spearman	t(N-2)	*p*-value
CD8+PD-1+ [%] & sCD200 [ng/ml]	-0.462	-3.165	0.003*
sCD200R [ng/ml] & WBC	-0.439	-2.969	0.005*
CD19+PD-1+ [%] & BAS	-0.419	-2.809	0.008*
CD4+CTLA-4+ [%] & WBC	-0.410	-2.735	0.010*
CD4+CD200+ [%] & sPD-1 [ng/ml]	-0.381	-2.509	0.017*
CD19+PD-L1+ [%] & MON	-0.376	-2.470	0.018*
CD4+CD200R+ [%] & sPD-L1 [ng/ml]	-0.367	-2.401	0.022*
CD19+CD86 [%] & sCD200R [ng/ml]	-0.363	-2.368	0.023*
CD19+CD200+ [%] & CD8+PD-L1+ [%]	-0.339	-2.191	0.035*
CD19+CD200R+ [%] & CD8+ CTLA-4+ [%]	-0.331	-2.132	0.040*
CD4+CD86+ [%] & EOS	-0.324	-2.084	0.044*
sPD-1 [ng/ml] & RBC	-0.322	-2.070	0.046*
CD4+CD200R+ [%] & sPD-1 [ng/ml]	-0.317	-2.030	0.050*
CD8+CD200+ [%] & CD8+ CTLA-4+ [%]	0.328	2.113	0.041*
CD4+ PD-L1+ [%] & PLT	0.329	2.117	0.041*
CD4+PD-1+ [%] & PLT	0.332	2.139	0.039*
CD19+CTLA-4+ [%] & MON	0.333	2.148	0.038*
CD4+PD-1+ [%] & IgG	0.338	2.187	0.035*
sCTLA-4 [ng/ml] & PLT	0.348	2.255	0.030*
CD8+ PD-1+ [%] & sCD86 [ng/ml]	0.348	2.258	0.030*
CD8+CD200R+ [%] & CD8+ PD-L1+ [%]	0.350	2.273	0.029*
CD4+PD-1+ [%] & CD8+PD-1+ [%]	0.361	2.356	0.024*
CD4+PD-1+ [%] & sCD86 [ng/ml]	0.363	2.372	0.023*
CD19+CTLA-4+ [%] & sPD-1 [ng/ml]	0.364	2.375	0.023*
CD4+CD200R+ [%] & HGB	0.370	2.422	0.020*
CD19+CD86 [%] & NEU	0.373	2.444	0.019*
sPD-L1 [ng/ml] & sCTLA-4[ng/ml]	0.378	2.480	0.018*
sCD200 [ng/ml] & sCTLA-4 [ng/ml]	0.381	2.506	0.017*
CD8+ CD200R+ [%] & CD8+ CTLA-4+ [%]	0.389	2.569	0.014*
sPD-L1 [ng/ml] & PLT	0.396	2.626	0.012*
CD19+PD-1+ [%] & CD19+PD-L1+ [%]	0.399	2.646	0.012*
CD4+ PD-L1+ [%] & IgA	0.412	2.747	0.009*
sCD200R [ng/ml] & sCTLA-4 [ng/ml]	0.420	2.815	0.008*
CD8+CD200R+ [%] & RBC	0.423	2.840	0.007*
CD4+CD200R+ [%] & RBC	0.462	3.171	0.003*
sCD200R [ng/ml] & sPD-1 [ng/ml]	0.484	3.361	0.002*
sCD200R [ng/ml] & EOS	0.498	3.495	0.001*

sPD-L1 [ng/ml] & sPD-1 [ng/ml]	0.498	3.495	0.001*
CD4+CD200+ [%] & LYM	0.553	4.035	0.000*
sCTLA-4 [ng/ml] & sPD-1 [ng/ml]	0.669	5.468	0.000*

* Statistically significant results

Supplementary Table S6. Correlation analysis of the tested parameters of the immune system and peripheral blood morphology and biochemistry in patients with CLL

A pair of variables	R Spearman	t(N-2)	p-value
CD8+ PD-1+ [%] & IgG	-0.513	-3.684	0.001*
CD4+CD200+ [%] & HGB	-0.499	-3.546	0.001*
CD19+CD200+ [%] & CD19+CTLA-4+ [%]	-0.466	-3.244	0.002*
CD19+CD200+ [%] & CD8+CD200R+ [%]	-0.429	-2.925	0.006*
CD4+CD200+ [%] & RBC	-0.427	-2.914	0.006*
CD19+CTLA-4+ [%] & LYM	-0.422	-2.873	0.007*
CD4+ PD-L1+ [%] & sCD200R [ng/ml]	-0.422	-2.867	0.007*
CD4+PD-L1+ [%] & sCD86 [ng/ml]	-0.390	-2.615	0.013*
CD19+CTLA-4+ [%] & CD4+CTLA-4+ [%]	-0.389	-2.605	0.013*
CD8+PD-1+ & sCD200 [ng/ml]	-0.384	-2.564	0.014*
CD8+ PD-1+ [%] & WBC	-0.379	-2.527	0.016*
CD19+PD-L1+ [%] & PLT	-0.376	-2.498	0.017*
CD8+CD200+ [%] & WBC	-0.374	-2.487	0.017*
CD4+CTLA-4+ [%] & IgA	-0.361	-2.383	0.022*
CD8+ PDL-1+ [%] & sCD200R [ng/ml]	-0.357	-2.356	0.024*
sCTLA-4 [ng/ml] & sCD86 [ng/ml]	-0.356	-2.347	0.024*
CD19+CD86 [%] & sCD86 [ng/ml]	-0.331	-2.164	0.037*
CD8+CD200R+ [%] & CD4+ CTLA-4+ [%]	-0.320	-2.080	0.044*
CD8+PD-1+ [%] & sPD-L1 [ng/ml]	-0.315	-2.049	0.047*
CD19+CTLA-4+[%] & IgM	0.314	2.037	0.049*
CD8+CD200R+ [%] & IgA	0.319	2.078	0.045*
CD4+CD86+ [%] & sCD86 [ng/ml]	0.325	2.120	0.041*
sCD200R [ng/ml] & WBC	0.327	2.130	0.040*
CD4+ CD200R+ [%] & CD8+ CD200R+ [%]	0.328	2.142	0.039*
CD19+CD200+ [%] & sCD200R [ng/ml]	0.329	2.144	0.038*
sPD-L1 [ng/ml] & sCD86 [ng/ml]	0.345	2.266	0.029*
CD8+CD200+ [%] & CD8+CD86+ [%]	0.355	2.343	0.024*
CD8+ CD200+ [%] & CD8+CTLA-4+ [%]	0.360	2.382	0.022*
CD19+CD200+ [%] & LYM	0.375	2.493	0.017*
CD19+CD200+[%] & CD4+ CTLA-4+ [%]	0.376	2.504	0.017*
sCD200R [ng/ml] & EOS	0.390	2.611	0.013*
sCD86 [ng/ml] & sCD200R [ng/ml]	0.397	2.663	0.011*
CD4+CD86+ [%] & HGB	0.397	2.669	0.011*

CD19+CD200R+ [%] & IgM	0.402	2.707	0.010*
CD4+CD86+ [%] & RBC	0.421	2.863	0.007*
CD8+CTLA-4+ [%] & BAS	0.426	2.899	0.006*
CD19+CD200R+ [%] & MON	0.426	2.900	0.006*
CD4+CD200+ [%] & sCD200R [ng/ml]	0.434	2.969	0.005*
CD4+PD-1+ [%] & CD8+PD-1+ [%]	0.441	3.029	0.004*
CD4+CD200+ [%] & sPD-1 [ng/ml]	0.442	3.039	0.004*

* Statistically significant results

Supplementary Materials Table S7. ROC curve analysis

Parameter		CLL vs. HV	CVID vs. HV	CLL vs. CVID
CD4+PD-1+[%]	AUC	1,000	1,000	0,598
	SE	0,000	0,000	0,064
	-95%	1,000	1,000	0,472
	+95%	1,000	1,000	0,724
	z statistic	-	-	1,522
	p value	0,000	0,000	0,128
CD8+PD-1+[%]	AUC	1,000	1,000	0,529
	SE	0,000	0,000	0,066
	-95%	0,000	1,000	0,401
	+95%	0,000	1,000	0,658
	z statistic	-	0,000	0,445
	p value	0,000	0,000	0,656
CD19+PD-1+[%]	AUC	1,000	1,000	0,969
	SE	0,000	0,000	0,018
	-95%	1,000	1,000	0,933
	+95%	1,000	1,000	1,000
	z statistic	-	-	25,905
	p value	0,000	0,000	0,000
CD4+PD-L1+[%]	AUC	0,97	0,952	0,591
	SE	0,018	0,026	0,064
	-95%	0,934	0,901	0,465
	+95%	1,000	1,000	0,717
	z statistic	25,812	17,352	1,416
	p value	0,000	457,495	0,157
CD8+PD-L1+[%]	AUC	0,841	0,794	0,571
	SE	0,051	0,056	0,065
	-95%	0,741	0,684	0,444
	+95%	0,941	0,903	0,697
	z statistic	6,688	5,260	1,090
	p value	0,000	0,000	0,275
CD19+PD-L1+[%]	AUC	0,976	0,956	0,584
	SE	0,014	0,021	0,064
	-95%	0,947	0,914	0,458

	+95%	1,000	0,998	0,710
	z statistic	33,136	21,36	1,304
	p value	0,000	0,000	0,192
CD4+CTLA-4+[%]	AUC	0,994	0,993	0,621
	SE	0,006	0,006	0,064
	-95%	0,983	0,981	0,496
	+95%	1	1,000	0,745
	z statistic	82,133	82,526	1,892
	p value	0,000	0,000	0,058
CD8+CTLA-4+[%]	AUC	1	0,999	0,706
	SE	0	0,001	0,061
	-95%	1	0,997	0,586
	+95%	1	1,000	0,825
	z statistic	-	457,495	3,371
	p value	0	0,000	0,001
CD19+CTLA-4+[%]	AUC	0,728	0,969	0,124
	SE	0,059	0,019	0,040
	-95%	0,613	0,932	0,046
	+95%	0,843	1,000	0,202
	z statistic	3,893	24,535	-9,446
	p value	0,000	0,000	0,000
CD4+CD86+[%]	AUC	1,000	0,999	0,473
	SE	0,000	0,001	0,066
	-95%	1,000	0,997	0,344
	+95%	1,000	1,000	0,602
	z statistic	-	457,495	-0,414
	p value	0,000	0,000	0,067
CD8+CD86+[%]	AUC	0,855	0,995	0,552
	SE	0,040	0,005	0,066
	-95%	0,776	0,985	0,421
	+95%	0,934	1,000	0,682
	z statistic	8,817	46,648	0,777
	p value	0,000	0,000	0,4374
CD19+CD86+[%]	AUC	1,000	0,990	0,013
	SE	0,000	0,010	0,009
	-95%	1,000	0,969	-0,004
	+95%	1,000	1,000	0,031
	z statistic	-	46,648	-53,185
	p value	0,000	0,000	0,000
CD4+CD200R+[%]	AUC	0,904	0,344	0,888
	SE	0,036	0,064	0,037
	-95%	0,832	0,219	0,816
	+95%	0,975	0,469	0,961
	z statistic	11,098	-2,452	10,487
	p value	0,000	0,014	0,000
CD8+CD200R+[%]	AUC	0,951	0,859	0,697
	SE	0,026	0,040	0,060
	-95%	0,9	0,780	0,579
	+95%	1	0,937	0,815
	z statistic	17,362	8,948	3,279
	p value	0,000	0,000	0,001
CD19+CD200R+[%]	AUC	0,143	0,030	0,697
	SE	0,043	0,016	0,059
	-95%	0,058	-0,002	0,583

	+95%	0,227	0,062	0,812
	z statistic	-8,253	-28,886	3,369
	p value	0,000	0,000	0,001
CD4+CD200+[%]	AUC	1,000	1,000	0,666
	SE	0,000	0,000	0,063
	-95%	1,000	1,000	0,544
	+95%	1,000	1,000	0,789
	z statistic	-	-	2,657
	p value	0,000	0,000	0,008
CD8+CD200+[%]	AUC	0,997	0,958	0,613
	SE	0,004	0,021	0,064
	-95%	0,990	0,917	0,487
	+95%	1,000	1,000	0,740
	z statistic	138,359	21,736	1,764
	p value	0,000	0,000	0,077
CD19+CD200+[%]	AUC	0,998	0,868	0,935
	SE	0,002	0,043	0,027
	-95%	0,993	0,784	0,882
	+95%	1,000	0,952	0,987
	z statistic	211,089	8,571	16,225
	p value	0,000	0,000	0,000
Serum concentration sPD-1	AUC	1,000	1,000	1,000
	SE	0,000	0,000	0,000
	-95%	1,000	1,000	1,000
	+95%	1,000	1,000	1,000
	z statistic	-	-	-
	p value	0,000	0,000	0,000
Serum concentration sPD-L1	AUC	1,000	1,000	1,000
	SE	0,000	0,000	0,000
	-95%	1,000	1,000	1,000
	+95%	1,000	1,000	1,000
	z statistic	-	-	-
	p value	0,000	0,000	0,000
Serum concentration sCTLA-4	AUC	1,000	1,000	0,907
	SE	0,000	0,000	0,031
	-95%	1,000	1,000	0,846
	+95%	1,000	1,000	0,968
	z statistic	-	-	13,054
	p value	0,000	0,000	0,000
Serum concentration sCD86	AUC	1,000	0,999	0,000
	SE	0,000	0,002	0,000
	-95%	1,000	0,995	0,000
	+95%	1,000	1,000	0,000
	z statistic	-	281,334	0,000
	p value	0,000	0,000	0,000
Serum concentration sCD200R	AUC	1,000	1,000	0,998
	SE	0,000	0,000	0,002
	-95%	1,000	1,000	0,993
	+95%	1,000	1,000	1,000
	z statistic	-	-	205,874
	p value	0,000	0,000	0,000
Serum concentration sCD200	AUC	1,000	1,000	0,532
	SE	0,000	0,000	0,066
	-95%	1,000	1,000	0,404

	+95%	1,000	1,000	0,661
	z statistic	-	-	0,492
	p value	0,000	0,000	0,622
